# Structural basis for ineffective T-cell responses to MHC anchor residue-improved “heteroclitic” peptides

**DOI:** 10.1002/eji.201445114

**Published:** 2014-12-28

**Authors:** Florian Madura, Pierre J Rizkallah, Christopher J Holland, Anna Fuller, Anna Bulek, Andrew J Godkin, Andrea J Schauenburg, David K Cole, Andrew K Sewell

**Affiliations:** Division of Infection and Immunity, Cardiff University School of MedicineHeath Park, Cardiff, UK

**Keywords:** Cross-reactivity, Crystal structure, MART-1, Melan-A, Melanoma, Peptide-major histocompatibility complex, Surface plasmon resonance, T-cell, TCR

## Abstract

MHC anchor residue-modified “heteroclitic” peptides have been used in many cancer vaccine trials and often induce greater immune responses than the wild-type peptide. The best-studied system to date is the decamer MART-1/Melan-A_26–35_ peptide, EAAGIGILTV, where the natural alanine at position 2 has been modified to leucine to improve human leukocyte antigen (HLA)-A*0201 anchoring. The resulting ELAGIGILTV peptide has been used in many studies. We recently showed that T cells primed with the ELAGIGILTV peptide can fail to recognize the natural tumor-expressed peptide efficiently, thereby providing a potential molecular reason for why clinical trials of this peptide have been unsuccessful. Here, we solved the structure of a TCR in complex with HLA-A*0201-EAAGIGILTV peptide and compared it with its heteroclitic counterpart, HLA-A*0201-ELAGIGILTV. The data demonstrate that a suboptimal anchor residue at position 2 enables the TCR to “pull” the peptide away from the MHC binding groove, facilitating extra contacts with both the peptide and MHC surface. These data explain how a TCR can distinguish between two epitopes that differ by only a single MHC anchor residue and demonstrate how weak MHC anchoring can enable an induced-fit interaction with the TCR. Our findings constitute a novel demonstration of the extreme sensitivity of the TCR to minor alterations in peptide conformation.

## Introduction

CD8^+^ T cells can exploit the major histocompatibility complex class I (MHCI) peptide presentation pathway to interrogate the cellular proteome and identify aberrant gene expression within cancer cells. These cytotoxic cells then eliminate tumor cells directly using an arsenal of granule-relayed and receptor-mediated cell death machinery; properties that have made CD8^+^ T cells an attractive target for therapeutic cancer vaccination [1]. An effective CD8^+^ T-cell response requires that the cell surface expressed TCR binds to its cognate peptide-MHCI (pMHCI) molecule with sufficient affinity/duration to induce cellular activation [2, 3]. We have demonstrated that TCRs bind more weakly to cancer epitopes compared to pathogenic epitopes [4, 5]. This important difference probably arises because most cancer epitopes are derived from self-molecules. Thus, potential cancer-specific T-cell clones with higher affinity TCRs are probably negatively selected against during the thymic auditioning process that has evolved to limit autoimmunity. Effective T-cell peptide epitopes are also required to form a stable interaction with MHCI [6]. The MHCI peptide-binding groove has several chemically distinct-binding pockets (termed A–F) [7–9]. Interaction between peptide and MHCI is principally governed by primary peptide anchor residues that are generally located toward the N- and C-terminus (MHCI-binding pockets B and F). This binding modality allows most MHCI molecules to present peptides of differing lengths by accommodating the extra amino acids as a central “bulge” within the binding groove [10, 11]. The extreme diversity of human MHCI genes [12] is focused upon these binding pockets so that different MHCIs have distinct optimal peptide-binding parameters, allowing them to interact with, and present, different subsets of peptides to T cells. The majority of cancer peptide epitopes that have been described do not contain optimal primary peptide anchor residues and many studies have focused upon modifying these residues (generating so called “heteroclitic” peptides) to improve the stability of these cancer epitopes at the cell surface [1, 13–16]. Because the TCR generally focuses on the central portion of the peptide (the so called “peptide bulge”) during antigen recognition [17, 18], modification at buried terminal amino acids were not thought to alter TCR binding. However, we have recently shown that the TCR can detect these changes in peptide sequence [19]. This fundamental observation is critical to the development of MHC anchor modified peptides, because vaccination using these reagents could lead to the targeting of T cells that can recognize the modified peptide, but not the natural peptide present on the surface of tumors. This observation could be a central factor in explaining why many clinical trials using heteroclitic peptides to treat cancer have failed.

Our previous study [19] focused on an HLA-A*0201 restricted Melan-A/MART-1_26–35_ (EAAGIGILTV) antigen (A2-EAA) [20]. The anchor-residue-modified heteroclitic version of this peptide, E**L**AGIGILTV (A2-ELA), is the most commonly described human tumor-related MHCI-restricted epitope in the current literature and has been shown to induce a far greater expansion of T cells when used in vaccination trials compared to A2-EAA [21, 22]. Despite A2-EAA inducing less than half of the response seen with A2-ELA, T cells primed with the natural antigen were found to have stronger tumor reactivity [23]. Analysis of the TCRs selected after vaccination of melanoma patients with these two peptides revealed subtle differences in the repertoire [24]. Our own clonotypic analysis of in vitro-expanded T-cell populations also showed that distinct T-cell clonotypes were generated depending on whether A2-ELA, or A2-EAA was used [19]. Molecular analysis highlighted that, although A2-ELA is more stable than A2-EAA, T cells can distinguish between these variants in cellular assays [19]. The MEL5 TCR bound to A2-ELA relatively weakly (K_D_ = 17–18 μM) [4, 19, 25] by surface plasmon resonance (SPR) compared to A2-EAA (K_D_ = 6.4 μM) [19]. Collectively, these findings could represent a major caveat to using A2-ELA for therapeutic melanoma vaccination because it can prime T cells that may not then respond optimally to natural tumor epitope.

Here, we set out to establish the molecular mechanism by which a TCR could distinguish between A2-EAA and A2-ELA. The atomic structures of these peptides in complex with HLA*0201 have been solved previously [26]. Both peptides adopt an identical bulged conformation, revealing no obvious mechanism for how a TCR could discriminate between them. The crystallographic structures of DMF4 and DMF5 [27], the first TCRs used in TCR gene transfer and adoptive cell therapy for cancer, and MEL5 TCR [25] in complex with the A2-ELA have been solved. To date there is no atomic structure of the natural A2-EAA peptide in complex with a TCR. Here, we fill this important knowledge gap by solving the atomic structure of the MEL5-A2-EAA complex and performing an in-depth biophysical analysis to determine the molecular mechanism for altered T-cell recognition between A2-ELA and the tumor expressed A2-EAA antigen by the MEL5 T cell. These data demonstrate the extreme sensitivity of the TCR to minor conformational changes in the peptide, extend our understanding of CD8^+^ T-cell recognition of a prominent tumor target and have important implications for the design of altered peptide ligands for use in cancer vaccination.

## Results

### MEL5 binds to A2-EAA with a distinct thermodynamic signature

**Figure 1:**
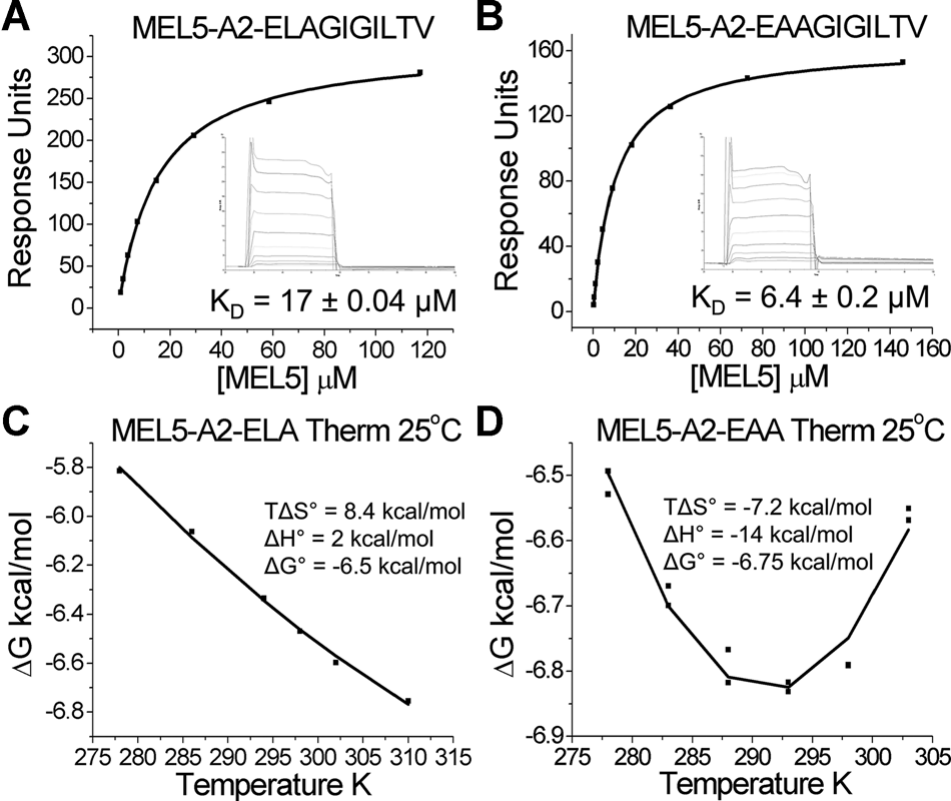
Thermodynamic signature of the MEL5-A2-EAA interaction. Binding affinity and thermodynamic analyses of the MEL5-A2-EAA interaction was performed using SPR. (A, B) The binding affinity of MEL5 for (A) A2-ELA and (B) A2-EAA were reproduced from [19, 25] by equilibrium analysis. (C) Thermodynamic analysis of the MEL5-A2-ELA interaction were reproduced from [25]. (D) Thermodynamic analysis of the interaction between MEL5 and A2-EAA was determined by SPR. K_D_s were measured in triplicate at 5, 10, 15, 20, 25, and 30°C with ten serial dilutions of MEL5 (Supporting Information Fig. 1); representative data from a single experiment is plotted. HLA-A*0201-ILAKFLHWL was used as a negative control surface on flow cell 1. The binding free energies (ΔG = RT ln K_D_) were plotted against temperature and the thermodynamic parameters (ΔH° and TΔS°) were calculated according to the nonlinear van't Hoff equation (RT ln K_D_ = ΔH° – TΔS° + ΔCp°(T - T_0_) – TΔCp° ln (T/T_0_)).

The affinity of MEL5 for A2-ELA and A2-EAA has been described previously [19] and reproduced here (Fig.1A and B). These data demonstrate that, although the modification to this peptide is located at peptide anchor residue 2 that does not make major interactions with the MEL5 TCR [25], the affinity of the MEL5-A2-EAA interaction is more than twice as strong as the MEL5-A2-ELA interaction [19]. This distinction has important implications for the use of the heteroclitic A2-ELA peptide in vaccination as previously described [19, 23, 24]. The structures of A2-ELA (PDB: 1JF1) and A2-EAA (PDB: 2GT9) provided no obvious reason for why a TCR could distinguish between them [26]. In order to determine the mechanism for how the MEL5 TCR bound to these peptides with over twofold difference in affinity, we performed a thermodynamic analyses of the MEL5-A2-EAA interaction and compared it to data for A2-ELA [25] (Fig.1C). These data demonstrated that MEL5 bound to A2-ELA with favorable entropy (TΔS° = 8.4 kcal/mol) and unfavorable enthalpy (ΔH° = 2 kcal/mol), suggesting that a transition from order to disorder, possibly through the expulsion of ordered water molecules, was the major driving force during binding. Surprisingly, and in contrast to the MEL5-A2-ELA interaction, the MEL5-A2-EAA interaction was enthalpically driven (ΔH° ∼ –14 kcal/mol) and entropically unfavorable (TΔS° ∼ –7.2 kcal/mol) (Fig.1D and Supporting Information Fig. 1), similar to that observed for other TCR-pMHC interactions [28]. These findings demonstrate a distinct energetic footprint for the MEL5-A2-EAA interaction, suggesting that a net formation of new electrostatic interactions drove the formation of the complex.

### MEL5 binds to A2-ELA and A2-EAA with similar conformation

**Figure 2:**
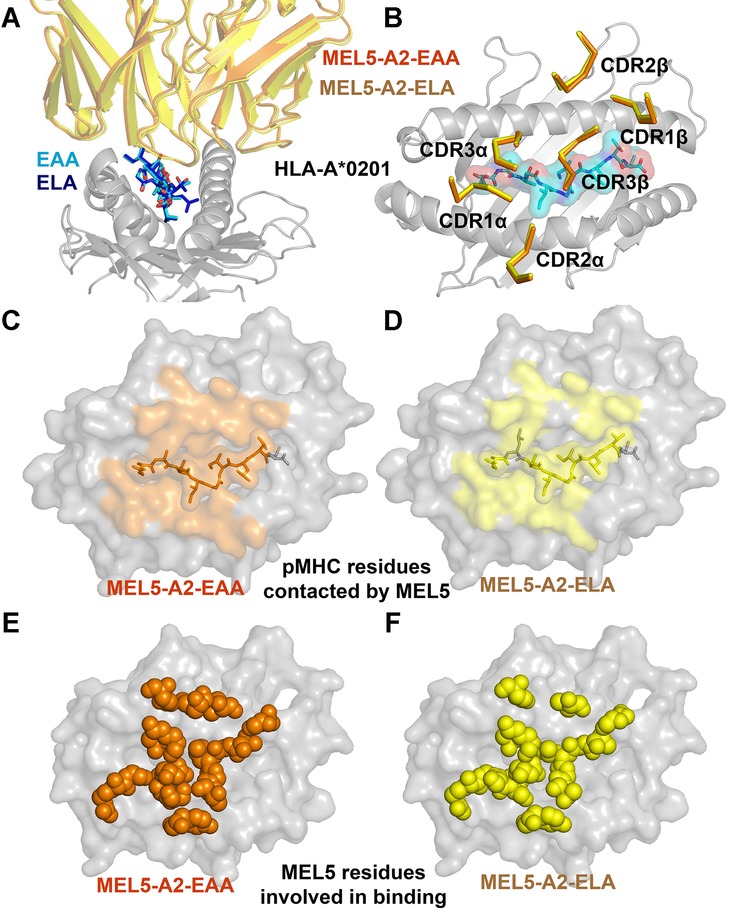
MEL5 utilizes an identical general binding mode to A2-EAA and A2-ELA. The MEL5-A2-EAA complex structure was solved with molecular replacement using PHASER [47]. The model sequence was adjusted with COOT [48] and the model refined with REFMAC5. (A) Superposition of the MEL5 V domain interacting with either A2-EAA (orange cartoon) or A2-ELA [25] (yellow cartoon). (B) Superposition of the MEL5 CDR loops interacting with either A2-EAA (orange cartoon) or A2-ELA (yellow cartoon). (C, D) MHC residues contacted by the MEL5 TCR are shown interacting with either (C) A2-EAA (orange surface) or (D) A2-ELA (yellow surface). (E, F) TCR residues used during the interaction with either (E) A2-EAA (orange balls) or (F) A2-ELA (yellow balls). Data shown were generated from one crystal.

In order to further understand the mechanism underlying the difference in T-cell recognition of the natural versus the heteroclitic MART-1/Melan-A_26–35_ peptide, we solved the crystal structure of MEL5 complexed to A2-EAA (Table1). The MEL5 TCR bound in a canonical diagonal docking mode at a crossing angle of ∼47°, with shape complementarity and buried surface area within the range described for other published TCR-pMHC interactions (Table2) [18, 29]. Superposition of the MEL5-A2-ELA and MEL5-A2-EAA structures, using the MHC molecule to align, demonstrated a similar-binding conformation (Fig.2A) with the TCR CDR loops positioned identically over the MHC-binding groove (Fig.2B). The contact footprint of the TCR over both surfaces was also similar, but not identical (Fig.2C and D), with MEL5 contacting more residues on the surface of A2-EAA than A2-ELA. Concordantly, the TCR residues utilized for binding were very similar in each case (Fig.2E and F), but again more TCR residues were involved in binding to A2-EAA than A2-ELA. These data demonstrate that the molecular mechanism explaining the difference in TCR-binding affinity and thermodynamics was not due to a large difference in binding orientation of the MEL5 TCR and warranted a more detailed investigation of the binding interface.

**Table 1:** Data collection and refinement statistics (molecular replacement)

Dataset statistics	MEL5-A2-EAA
PDB	4QOK
Space group	P4_1_
Unit cell parameters (Å)	a = 121.40, b = 121.40, c = 82.32
Radiation source	DIAMOND I04–1
Wavelength (Å)	0.9173
Resolution (Å)	41.03–3.00 (3.08–3.0)
Unique reflections	22 733 (1726)
Completeness (%)	99.9 (99.9)
Multiplicity	7.5 (7.9)
I/Sigma(I)	12.8 (3.1)
Rmerge	0.122 (0.820)
Refinement statistics	
No reflections used	22 556
No reflections in Rfree set	1219
Rcryst (no cutoff) (%)	20.3
Rfree (%)	26.2
RMSD from ideal geometry	
Bond lengths (Å)	0.013
Bond angles (°)	1.452
Mean B value (Å^2^)	70.8
Wilson B-factor (Å^2^)	78.4
ESU based on maximum likelihood (Å)	0.31
ESU for B values based on maximum likelihood (Å^2^)	16.3

One crystal was used for data collection.

Number in parentheses indicate the outer-resolution shell.

**Table 2:** Detailed analysis of MEL5-A2-EAA versus MEL5-A2-ELA structures

	MEL5-A2-EAA	MEL5-A2-ELA [25]
Total No. TCR-pMHC contactsa)	12/104	7/86
No. V_α_ contactsa)	7/49	4/44
No. V_β_ contactsa)	5/55	3/42
No. TCR-peptide contactsa)	6/39	3/33
No. TCR-MHC contactsa)	6/65	4/53
BSAb) (Å^2^)	2366	2528
SCc) (%)	59.9/74.5/64	73.1/56/66.6
Crossing angle (°)	46.9	42.6

a) Number of hydrogen bonds (H-bond) (≤3.4Å) and salt bridges (≤3.4Å)/van der Waals (vdW) (3.2–4Å) contacts calculated with the CONTACT program from the CCP4 package.

b) Buried surface area (BSA) (Å^2^) calculated with PISA program from the CCP4 package.

c) Shape complementarity (SC) (%) of TCR-MHC/TCR-peptide/TCR-pMHC calculated with SC program from the CCP4 package.

### N-terminal peptide flexibility enables additional contacts between A2-EAA and the MEL5 TCR

**Figure 3:**
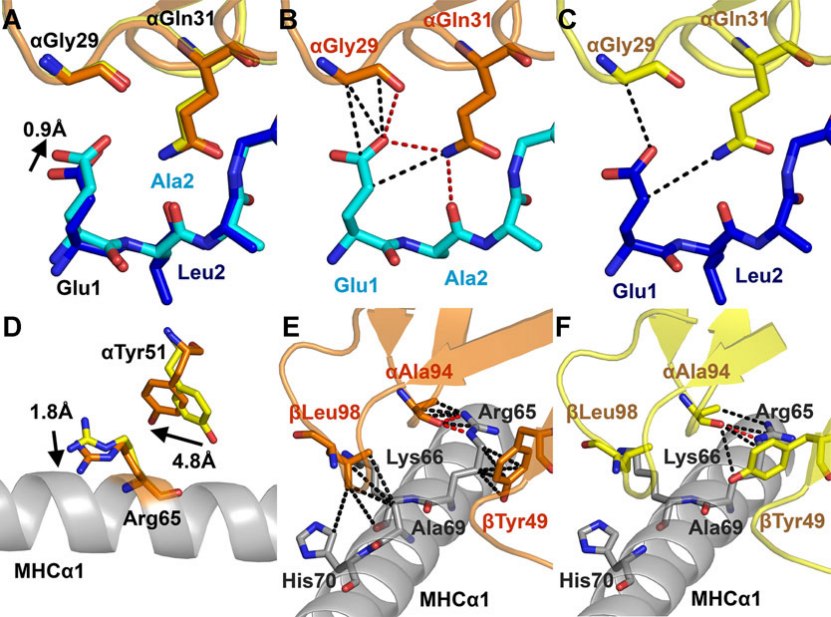
Altered anchoring of peptide to MHC changes TCR contacts. The MEL5-A2-EAA complex structure was analyzed using the program CONTACT from the CCP4 software suite and graphical representation was prepared using PYMOL. (A) The position of the EAA peptide (cyan sticks) compared to the ELA peptide (blue sticks) in complex with the MEL5 TCR (MEL5-A2-EAA; orange and MEL5-A2-ELA; yellow sticks) is shown. (B) Hydrogen bonds (red dotted lines) and vdW contacts (black dotted lines) are shown between the EAA peptide (cyan sticks) and the MEL5 TCR CDR1 loop (orange sticks). (C) Hydrogen bonds and vdW contacts are shown between the ELA peptide (blue sticks) and the MEL5 TCR CDR1 loop (yellow sticks). (D) The position of MHCα1 (gray cartoon) residue Arg65 and MEL5 α chain residue Tyr51 in the MEL5-A2-EAA complex (orange sticks) compared to the MEL5-A2-ELA complex (yellow sticks). (E) Hydrogen bonds and vdW contacts are shown between the MEL5 TCR (orange cartoon) and the MHCα1 helix (gray cartoon) in the MEL5-A2-EAA complex. (E) Hydrogen bonds and vdW contacts are shown between the MEL5 TCR (yellow cartoon) and the MHCα1 helix (gray cartoon) in the MEL5-A2-ELA complex. Data shown were generated from one crystal.

In both the MEL5-A2-EAA and MEL5-A2-ELA structures, the majority of the contacts were made in the central region of the peptide (residues P4–P7) (Supporting Information Table 1). The MEL5 Vα domain contacted peptide residues P1–P5, predominantly through the TCR CDR1α loop (Fig.3, Supporting Information Tables 1 and 2). This is distinct from the generally observed TCR-peptide interaction that is normally dominated through the TCR CDR3 loops [30]. The MEL5 Vβ domain contacted residues P5–P9, with TCR CDR3β aligning alongside the C-terminal half of the peptide, as seen with the DMF4 TCR [27]. Although all three MHC restriction triad residues [10] (Arg65, Ala69, and Gln155) were involved in the interaction between MEL5 TCR and A2-ELA, only 2 (Arg65 and Gln155) formed bonds in the MEL5-A2-EAA complex (Supporting Information Tables 1 and 2). In agreement with the distinct thermodynamic signature (Fig.1), MEL5 made more contacts with A2-EAA (eight hydrogen bonds, four salt bridges, and 104 vdW interactions) than with A2-ELA (five hydrogen bonds, two salt bridge, and 104 vdW interactions). The greater number of bonds between MEL5 and A2-EAA also offers an explanation for the stronger affinity between these two molecules. Although the overall conformation of the two complexes was similar (Fig.2), on closer inspection it was evident that the N-terminus of the EAA peptide extended 0.9Å closer to the MEL5 TCR compared to the uncomplexed A2-ELA and A2-EAA structures [26] and the MEL5-A2-ELA complex (Fig.3A) [25]. This conformational distinction was possible because the smaller alanine side chain at position 2 in the EAA peptide made a less optimal interaction with the B pocket of the HLA A*0201 molecule, compared to the longer leucine side chain in the ELA peptide. This difference also explains our previous observation demonstrating that the A2-EAA complex is less stable than A2-ELA [19]. Thus, MEL5 was able to “pull” the N-terminus of the EAA peptide away from the MHC surface and make a different network of contacts compared to A2-ELA. Overall, MEL5 made three hydrogen bonds and four vdW interactions with EAA peptide residues Glu1 and Ala2, compared to only two vdW interactions with ELA peptide residues Glu1 and Leu2 (Fig.3B and C). In addition to the differences in peptide contacts, MEL5 also made distinct contacts with the MHC surface in the MEL5-A2-EAA complex. MHC residue Arg65 underwent a 1.8Å movement between the two complex structures, coupled with a 4.8Å movement in TCRα chain residue Tyr51 (Fig.3D). These movements enabled MEL5 residues αAla94 and βTyr49 to make two salt bridges and nine vdW interactions with MHC residue Arg65 (compared to one salt bridges and three vdW interactions for A2-ELA) and TCR residue βLeu98 to make five vdW contacts with MHC residues Ala69 and His70 (compared to none for A2-ELA) in the MEL5-A2-EAA complex (Fig.3E and F). These data show that small changes in peptide flexibility can lead to substantially different TCR contacts. These structural observations offer a novel molecular mechanism that explains the differences in TCR-binding affinity and T-cell recognition that we [19], and others [23], have previously observed between A2-EAA and A2-ELA.

## Discussion

The majority of tumor-derived MHCI-associated peptides that have been described as T-cell epitopes have suboptimal anchor residues [1]. This observation might be because self-antigens that bind tightly to MHC are presented at higher concentrations in the thymus, leading to stronger T-cell activation and negative selection. It is possible that tumor-derived peptides with poor MHC anchors exhibit reduced cell surface expression levels in the thymus, thereby generating weak T-cell activation and positive selection. This notion has also been suggested for a number of T-cell responses to autoantigens where the auto-peptide bound weakly to MHC [31, 32]. In addition, tumor-specific T cells generally have weakly binding TCRs [4, 5]. These factors combine with various immunosuppressive mechanisms within tumors to ensure that T-cell responses to neoplastic cells are often poor. To overcome this lack of immunogenicity, a number of anchor residue modified “heteroclitic” peptides have been developed where amino acids in the natural sequence that act as weak primary MHC anchors are substituted for optimal anchors [1, 13–16]. A requirement of these reagents is that they should not alter T-cell specificity, or the T cells selected with the heteroclitic peptide may not respond to the peptide expressed at the tumor cell surface in vivo. Consistent with this notion, previous studies have demonstrated the importance of designing heteroclitic peptides that are the closest structural mimics of the original wild-type peptide [33]. Because the TCR does not usually contact the anchor residues directly [18], the assumption has been that anchor residue modifications should not be detected by the TCR. However, we have recently shown that T cells can distinguish between peptides with modified anchors, and these heteroclitic peptides can activate a distinct T-cell population that does not recognize the unmodified tumor-expressed peptide [19]. Importantly, different efficacies and TCR repertoires have been observed between natural and heteroclitic mimitopes during cancer vaccine trials [23, 24] offering a potential explanation for why some of these vaccine trials have failed.

Here, we reveal the structural mechanism for changes in TCR specificity mediated by the commonly used heteroclitic peptide in the HLA-A*0201-restricted MART-1/Melan-A_26–35_ system. Several studies have shown that TCRs can distinguish between the natural HLA-A*0201-EAAGIGILTV antigen and the HLA-A*0201-E**L**AGIGILTV variant [19, 23, 24]. The structures of these two molecules have provided no obvious reason for why TCRs could distinguish between them [26]. We find that although the MEL5 TCR bound with an identical overall conformation to A2-EAA and A2-ELA, the weaker anchor residue at position 2 in the natural peptide enabled the TCR to “pull” the peptide away from the MHC peptide-binding groove. This small movement facilitated nine extra contacts with the peptide, and indirectly led to 14 extra contacts with the MHC surface compared to the MEL5-A2-ELA interaction. These extra bonds explain the stronger binding affinity for the MEL5-A2-EAA interaction and demonstrate the sensitivity of the TCR for detecting changes in the peptide, in line with our previous findings [34, 35]. Our previous thermodynamic experiments demonstrated that MEL5 bound to A2-ELA with favorable entropy and unfavorable enthalpy [25], suggesting that a transition from order to disorder, possibly through the expulsion of ordered water molecules, mediated the interaction. In contrast, MEL5 bound to A2-EAA with favorable enthalpy suggesting that, consistent with our structural observations, a net increase in electrostatic interactions drove binding. Thus, MEL5 bound to A2-EAA with a distinct energetic signature compared to A2-ELA (entropically favorable; order to disorder driven) that was enthalpically favorable, demonstrated by the increase in interactions we observed at the MEL5-A2-EAA interface, which mediated the stronger binding affinity compared to MEL5-A2-ELA. The observation that an increase in TCR-binding affinity is associated with a switch from favorable entropy to favorable enthalpy is consistent with findings from other studies [36].

In summary, we demonstrate that peptides with suboptimal anchor residues can be more flexible within the MHC-binding groove, thereby enabling them to form subtly different conformational motifs that have an effect on the fine specificity of the TCR. In the case of MEL5, we previously hypothesized that increased flexibility in the P1–P2 region of the peptide might allow for stronger, or even new, TCR-peptide contacts that could explain the enhanced TCR affinity and preferential antigen sensitivity of MEL5 for A2-EAA (K_D_ = 6.4 μM) compared with A2-ELA (K_D_ = 17–18 μM). The MEL5-A2-EAA structure confirms this hypothesis. These data constitute a novel demonstration of the extreme sensitivity of the TCR to minor alterations in peptide conformation, extending our understanding of the molecular intricacies of T-cell antigen recognition with attendant implications for the design of altered peptides for therapy. Future use of altered peptide ligands for breaking tolerance to cancer epitopes will require rigorous testing to ensure that these peptides skew the TCR repertoire that is primed toward beneficial clonotypes that have a strong reactivity toward the natural epitope [37–40].

## Materials and methods

### Generation of the MEL5 TCR

The MEL5 TCR was derived from the MEL5 CD8^+^ T-cell clone specific for HLA-A*0201 MART-1/Melan-A_26–35_ (EAAGIGILTV) [25].

### Cloning and expression

The MEL5 TCR α and MEL5 TCR β chains, HLA-A*0201 heavy chain and β2m sequences were cloned into the pGMT7 expression vector under the control of the T7 promoter as described previously [41]. The MEL5 TCR α and MEL5 TCR β chains, the HLA A*0201 α chain, and β2m were expressed separately in competent Rosetta DE3 *Escherichia coli* cells as described previously [42].

### Refolding and purification

Refolding was performed as previously described [4]. Briefly, for a 1 L MEL5 TCR refold, 30 mg of MEL5 α-chain was incubated at 37°C for 30 min with 10 mM DTT and added to refold buffer at 4°C (50 mM TRIS pH 8.1, 2 mM EDTA, 2.5 M urea, 6 mM cysteamine hydrochloride, and 4 mM cystamine). After 30 min, 30 mg MEL5 β-chain, also incubated at 37°C for 30 min with 10 mM DTT, was added. For a 1 L pMHCI refold, 30 mg HLA A*0201 α-chain was mixed with 30 mg β2m and 4 mg EAAGIGILTV peptide at 37°C for 30 min with 10 mM DTT. This mixture was then added to refold buffer at 4°C (50 mM TRIS pH 8.1, 2 mM EDTA, 400 mM L-arginine, 6 mM cysteamine hydrochloride, and 4 mM cystamine). The MEL5 TCR and A2-EAA refolds were mixed at 4°C for 1–3 h and dialyzed against 10 mM TRIS pH 8.1 until the conductivity of the refolds was <2 mS/cm. Refolded proteins were purified by ion exchange using a Poros50HQ™ column (GE Healthcare, Buckinghamshire, UK) and gel filtered into crystallization buffer (10 mM TRIS pH 8.1 and 10 mM NaCl) or BIAcore buffer (10 mM HEPES pH 7.4, 150 mM NaCl, 3 mM EDTA, and 0.005% v/v surfactant P20) using a Superdex200HR™ column (GE Healthcare, Buckinghamshire, UK).

### Protein crystallization

Crystals were grown at 18°C by vapor diffusion via the sitting drop technique as previously described [43]. For MEL5-A2-EAA, optimal crystals were obtained in TOPS [44] with 0.1 M Sodium cacodylate pH 6.5, 15% PEG 4000, and 15% glycerol.

### Structure determination and refinement

Diffraction data were collected at a beamline I0–2 at the Diamond Light Source, Oxford, using a Pilatus 2M detector, a QADSC detector or a Rayonix detector. Using a rotation method, 400 frames were recorded each covering 0.5° of rotation. Reflection intensities were estimated with the XIA2 package [45] and the data were scaled, reduced, and analyzed with SCALA and the CCP4 package [46]. The TCR-pMHC complex structure was solved with molecular replacement using PHASER [47]. The model sequence was adjusted with COOT [48] and the model refined with REFMAC5 [49]. Accession code PDB: 4QOK.

### pMHC biotinylation

Biotinylated pMHCs were prepared as described previously [4].

### Surface plasmon resonance analysis

Binding analysis was performed using a BIAcore T100™ equipped with a CM5 sensor chip as previously described [19]. MEL5 was purified and concentrated to ∼150 μM on the same day of surface plasmon resonance analysis to reduce the likelihood of TCR aggregation affecting the results. For equilibrium analysis, ten serial dilutions were prepared in triplicate for each sample and injected over the relevant sensor chips at 25°C. MEL5 was injected over the chip surface using kinetic injections at a flow rate of 45 μL/min using HLA-A*0201-ILAKFLHWL as a negative control surface. For the thermodynamics experiments, this method was repeated at the following temperatures: 5, 10, 15, 20, 25, and 30°C. Results were analyzed using BIA evaluation 3.1, Excel, and Origin 6.0 software. The equilibrium-binding constant (K_D_) values were calculated assuming a 1:1 interaction by plotting specific equilibrium-binding responses against protein concentrations followed by nonlinear least squares fitting of the Langmuir-binding equation. The thermodynamic parameters were calculated using the nonlinear van't Hoff equation (RT ln K_D_ = ΔH° – TΔS° + ΔCp°(T – T_0_) – TΔCp° ln (T/T_0_)) with T_0_ = 298 K.

## Abbreviations

pMHC: peptide-major histocompatibility complex
SPR: surface plasmon resonance
