# Supplementary material for: Structural basis for ineffective T-cell responses to MHC anchor residue-improved “heteroclitic” peptides
Source: Eur J Immunol. 2014 Dec 28;45(2):584–91. doi: 10.1002/eji.201445114 (PMC4357396; doi:10.1002/eji.201445114)
Supplement: Supplementary file 1 [file eji0045-0584-sd1.pdf]

European Journal of Immunology

Supporting Information

for

DOI 10.1002/eji.201445114

Florian Madura, Pierre J. Rizkallah, Christopher J. Holland, Anna Fuller, Anna Bulek, Andrew J. Godkin, Andrea J. Schauenburg, David K. Cole and Andrew K. Sewell

Structural basis for ineffective T-cell responses to MHC anchor residue-improved "heteroclitic" peptides

© 2014 The Authors. *European Journal of Immunology* Published by Wiley-VCH Verlag GmbH & Co. KGaA, Weinheim

Supporting Information

Structural basis for ineffective T-cell responses to MHC anchor residue-improved 'heteroclitic' peptides

Florian Madura[1], Pierre J. Rizkallah[1], Christopher J. Holland[1], Anna Fuller[1], Anna Bulek[1], Andrew J Godkin[1], Andrea A.J. Schauenburg[1], David K. Cole[1*] and Andrew K. Sewell[1*]

[1]Cardiff University School of Medicine, Heath Park, Cardiff, CF14 4XN, UK.
[*]These authors contributed equally to this study

Running title: Altered anchoring of peptide to MHC changes TCR binding

To whom correspondence should be addressed: Professor Andrew Sewell, E-mail: sewellak@cf.ac.uk. Tel: +442920687055, or Dr David Cole, E-mail: coledk@cf.ac.uk. Tel: +442920687006

Keywords: MART-1, Melan-A, melanoma, crystal structure, peptide-major histocompatibility complex (pMHC), surface plasmon resonance (SPR), T-cell, T-cell receptor (TCR), cross-reactivity.

Non-standard abbreviations: pMHC, peptide-major histocompatibility complex; RU, response unit; SPR, surface plasmon resonance.

Supplementary Table S1: MEL5-A2-EAA. 3.4Å cut-off for H-bonds, 3.4Å cut-off for salt bridges and 4Å cut-off for vdW.

Peptide	TCR	H-bonds (≤3.4Å)	vdW (3.2Å–4Å)
Glu1$^{O\varepsilon 2}$	αGly29^{O}	1	3
Glu1$^{O\varepsilon 2}$	αGln31$^{N\varepsilon 2}$	1 salt bridge	1
Ala2^{O}	αGln31$^{N\varepsilon 2}$	1	
Ala3	αGln31		1
Gly4^{N}	αGln31$^{O\varepsilon 1}$	1	6
Gly4	βLeu98		3
Ile5	αGln31		2
Ile5	αSer32		1
Ile5	αAsn92		1
Ile5	βLeu98		2
Ile5	βGly99		3
Gly6^{N}	βLeu98^{O}	1	2
Ile7	βGly97		2
Ile7^{O}	βLeu98^{N}	1	9
Leu8	βGly99		2
Thr9	βThr96		1
MHC	TCR	H-bonds (≤3.4Å)	vdW (3.2Å–4Å)
Gly62	αAla94		3
Arg65$^{N\varepsilon/NH2}$	αAla94^{O}	2	6
Arg65	αLys96		3
Arg65	βTyr49		6
Arg65^{NH1}	βGlu59$^{O\varepsilon 1/O\varepsilon 2}$	2 salt bridge	2
Lys66	αGln31		1
Lys66	βLeu98		1
Lys68	βTyr49		2
Ala69	βLeu98		4
His70	βLeu98		1
Gln72	βVal51		2
Thr73	βGly97		2
Val76	βAsn30		4
His151	αTyr51		1
Glu154	αTyr51		3
Gln155	αTyr51		5
Gln155	βGly99		2
Gln155$^{O\varepsilon 1}$	βThr100^{N}	1	5
Ala158	αTyr51		2
Tyr159	αGln31		3
Thr163	αGln31		3
Glu166$^{O\varepsilon 2}$	αArg28^{NH2}	1 salt bridge	2
Trp167	αArg28		1
Trp167	αGly29		1

Supplementary Table 2: MEL5-A2-ELA (1). 3.4Å cut-off for H-bonds, 3.4Å cut-off for salt bridges and 4Å cut-off for vdW.

Peptide	TCR	H-bonds (≤3.4Å)	vdW (3.2Å–4Å)
Glu1	αGly29		1
Glu1	αGln31		1
Ala3	αGln31		1
Ala3	βLeu98		1
$Gly4^{N}$	$\alpha Gln31^{O\varepsilon 1}$	1	5
$Gly4^{O}$	$\alpha Asn92^{N\delta 2}$	1	
Gly4	βLeu98		3
Ile5	αGln31		1
Ile5	αSer32		1
Ile5	αTyr51		1
Ile5	αAsn92		1
Ile5	βLeu98		2
Ile5	βGly99		6
Gly6	βGly99		2
$Ile7^{O}$	$\beta Leu98^{N}$	1	4
Ile7	βGly99		1
Leu8	βGly99		2
Thr9	βThr96		1
**MHC**	**TCR**	**H-bonds (≤3.4Å)**	**vdW (3.2Å–4Å)**
Gly62	αAla94		2
$Arg65^{N\varepsilon}$	$\alpha Ala94^{O}$	1	5
Arg65	αLys96		2
$Arg65^{NH1}$	$\beta Glu59^{O\varepsilon 1}$	1 salt bridge	1
Lys66	αGln31		1
Lys68	βVal51		1
Gln72	βVal51		3
Thr73	βGly97		2
Val76	βAsn30		3
Val76	βThr96		1
Glu154	αTyr51		3
Gln155	αTyr51		5
Gln155	βGly99		2
$Gln155^{O\varepsilon 1}$	$\beta Thr100^{N}$	1	7
Ala158	αTyr51		2
Tyr159	αGln31		4
Thr163	αGln31		4
$Glu166^{O\varepsilon 2}$	$\alpha Arg28^{NH2}$	1 salt bridge	2
Trp167	αGly29		3

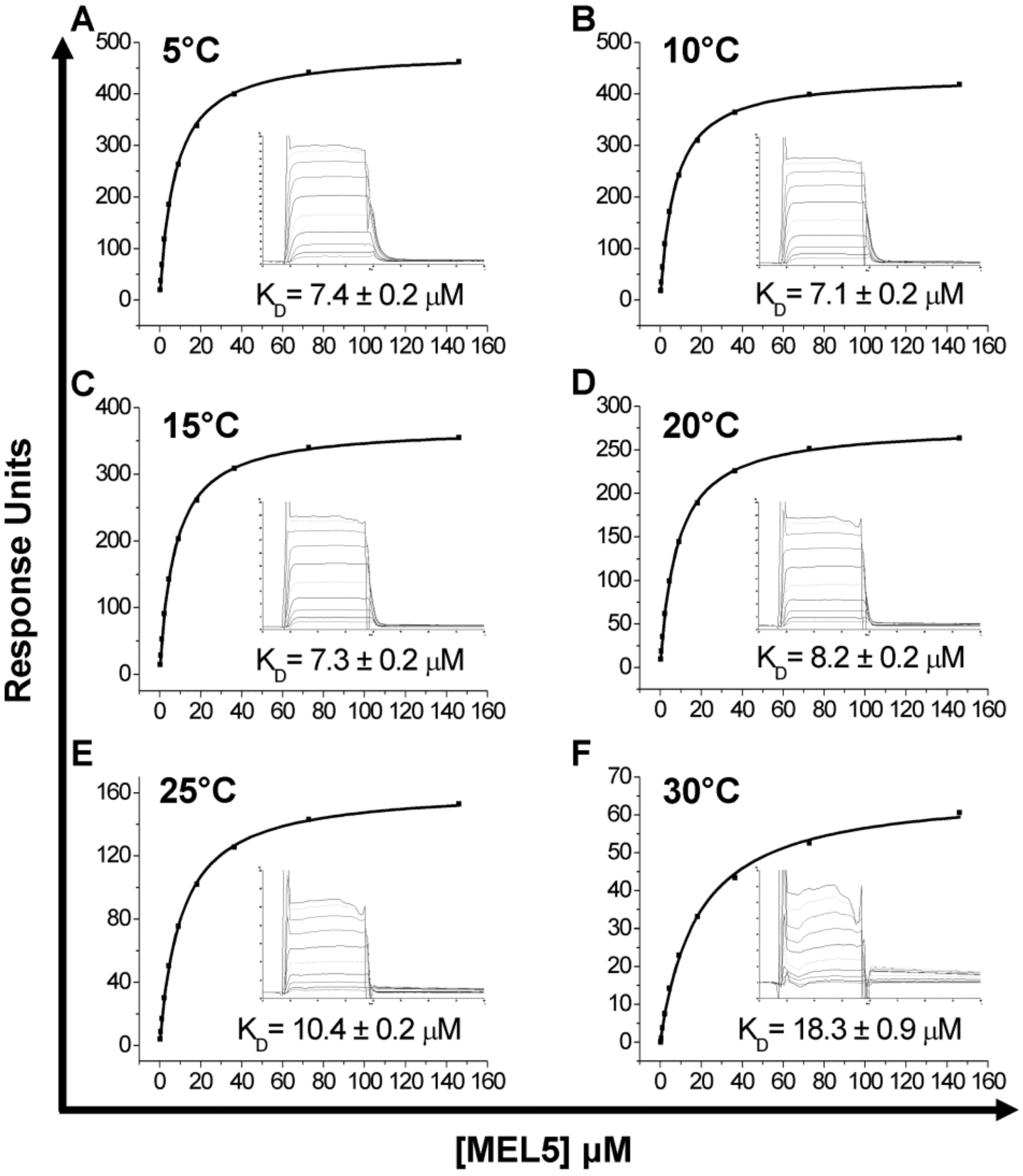

Supplementary Figure S1. Thermodynamic analysis of the MEL5 interaction with A2-EAA. Thermodynamic analysis of the MEL5-A2-EAA interaction was performed by SPR. Ten serial dilutions of MEL5 were measured in triplicate at **(A)** 5°C, **(B)** 10°C, **(C)** 15°C, **(D)** 20°C, **(E)** 25°C and **(F)** 30°C; representative data from one of these experiments is plotted. The equilibrium binding constant (K_D) values were calculated using a nonlinear curve fit (Langmuir binding equation $AB = (B \times AB_{max})/(K_D + B)$); mean plus SD values are shown. These data were used to fit thermodynamic parameters shown in **Figure 1.**
