# Supplementary material for: Structural basis for ineffective T-cell responses to MHC anchor residue-improved “heteroclitic” peptides
Source: Eur J Immunol. 2014 Dec 28;45(2):584–91. doi: 10.1002/eji.201445114 (PMC4357396; doi:10.1002/eji.201445114)
Supplement: Supplementary file 2 [file eji0045-0584-sd2.pdf]

Peer review correspondence

European Journal of Immunology

Structural basis for ineffective T-cell responses to MHC anchor residue-improved ‘heteroclitic’ peptides

Florian Madura, Pierre J. Rizkallah, Christopher J. Holland, Anna Fuller, Anna Bulek, Andrew J. Godkin, Andrea J. Schauenburg, David K. Cole and Andrew K. Sewell

Corresponding author: Andrew K. Sewell, Cardiff University School of Medicine, Heath Park, Cardiff, United Kingdom.

Review Timeline:	Submission date:	9 August 2014
	Editorial decision:	24 September 2014
	Revision received:	3 October 2014
	Accepted:	26 November 2014

Handling Executive Committee member: Prof. David Gray

Please note that the correspondence below does not include the standard editorial instructions regarding preparation and submission of revised manuscripts, only the scientific revisions requested and addressed.

First Editorial Decision – 24 September 2014

Dear Prof. Sewell,

Apologies for the delay in processing the review of your Manuscript ID eji.201445114 entitled "Structural basis for ineffective T-cell responses to MHC anchor residue improved ‘heteroclitic’ peptides", which you submitted to the European Journal of Immunology. There was a delay in sourcing two qualified reviewers and thereafter another delay in one of the reports. All opinions have now been received and the comments of the referees are included at the bottom of this letter.

Although the referees have recommended publication, some revisions to your manuscript have been requested. Therefore, I invite you to respond to the comments of the referees and revise your manuscript accordingly.

European Journal of Immunology

You should also pay close attention to the editorial comments included below. *In particular, please edit your figure legends to follow Journal standards as outlined in the editorial comments. Failure to do this will result in delays in the re-review process.*

If the revision of the paper is expected to take more than three months, please inform the editorial office. Revisions taking longer than six months may be assessed by new referees to ensure the relevance and timeliness of the data.

Once again, thank you for submitting your manuscript to European Journal of Immunology. We look forward to receiving your revision.

Yours sincerely,
Karen Chu

on behalf of Prof. David Gray

Dr. Karen Chu
Editorial Office
European Journal of Immunology
e-mail: ejied@wiley.com
www.eji-journal.eu

****

Reviewer: 1

Comments to the Author
The manuscript by Madura et al. addresses a clinically relevant (T cell epitopes for vaccine design) aspect of T cell biology - why heteroclitic peptides with optimised anchor residues (P2 in this example WT:EAAGIGILTV heteroclitic: ELAGIGILTV) can induce T cell responses which have altered recognition of the WT peptide despite both having the same TCR facing side-chains. The molecular basis of a well characterised example of this effect is explored here.

The data are comprehensive. The MEL5 TCR (raised against the WT peptide EAAGIGILTV) had higher affinity (~2x by SPR) for the WT peptide compared to the heteroclitic. Additionally, thermodynamic analysis revealed distinct energetic footprints, with the WT peptide being more typical (enthalpically driven).

European Journal of Immunology

This group previously solved the solved the MHC-peptide-TCR structure with the heteroclitic peptide, here they report the corresponding structure with the WT peptide. Comparison of the two (and the corresponding unliganded pMHC structures) reveals the WT peptide (but not the heteroclitic) is 'pulled' away from the peptide groove enabling additional interactions which explain the increased affinity over the heteroclitic peptide.

Overall, this work provides one mechanism whereby heteroclitic peptides (with enhanced MHC binding) may be differentially 'seen' by the TCR repertoire. Indeed this feature (peptide positioned 'pulled' away from the MHC) of TCR recognition is, to my knowledge, has not been reported before adding another, intersting facet to TCR recognition.

Some additional discussion would improve the manuscript.

-If possible, some clarification on how TCR binding of EAA and ELA are thermodynamically different (entropy/enthalpy).

-As the 2 unliganded pMHC structures are very similar (peptide seated in groove) - what does this mean for MHC on the cell surface - is there wobble in/out of the groove? Does EAA also stimulate T cells which recognise this (dominant?) form of the complex? How do they account for the low reactivity of T cells raised to ELA to EAA? In this setting, EAA in the right conformation (seen in crystal structures) should be available on the cell surface. Is it also to do with ligand stability/density on the surface?

-In this example, EAA (used to raise MEL5) is the WT and ELA is the heteroclitic peptide. As the authors stress, it is patient responses raised to the heteroclitic peptide which can cross-react poorly with the WT potentially accounting for the poor clinical outcome. This study looks at the reverse situation - poor recognition of the heteroclitic peptide in comparison with the WT by a TCR raised against the WT. The authors should comment on this. Would it have been more useful to look at the former, problematic situation?

Minor point- Abstract: has the ELA peptide really been used in 'hundreds of studies' ? If not, 'many studies' better.

Reviewer: 2

Comments to the Author

European Journal of Immunology

The manuscript of Madura et al presents extremely convincing and novel structural and biophysical results regarding the inefficient recognition of heteroclitic peptides by TCRs. The study is very well performed and the results are in my opinion clear-cut. The manuscript is well written and to the point.

Besides some minor points regarding the presentation of the crystallography statistics (see below), and some references that should be added in order to enhance the readers understanding of this very sensitive recognition, I think that the quality of the presented work, and the importance of their implications renders this manuscript of high interest to the community.

The results obtained in this study about the differential thermodynamic signature used by the TCR for recognizing the MHC molecule in complex with slightly different peptides are important (lines 87-97) and should be compared and discussed in regards to the results included in the publication by Allerbring et al, EJI 2013.

Furthermore, the discussion section which is quite clear and precise should include a comment of the results presented in van Stipdonk et al, Cancer Research 2009 where similar results regarding the increased recognition of a heteroclitic version of the melanoma-associated gp100-derived epitope which is obtained only upon keeping the most profound structural mimicry and the altered peptide version.

Minor points.

The authors should add information about the highest resolution shell.

It is also important that a specific section entitled 'analysis of B factors' is added before the Wilson B factors. Here the authors must add the average B values for the model.

Finally the authors should provide a thorough explanation for the overall ESU based on maximum likelihood value.

<u>First revision – authors' response – 3 October 2014</u>

RESPONSE TO REVIEWER 1:

Reviewer 1 comment: The manuscript by Madura et al. addresses a clinically relevant (T cell epitopes for vaccine design) aspect of T cell biology - why heteroclitic peptides with optimised anchor residues (P2 in this example WT:EAAGIGILTV heteroclitic: ELAGIGILTV) can induce T cell responses which have altered recognition of the WT peptide despite both having the same TCR facing side-chains. The molecular basis of a well characterised example of this effect is explored here.

European Journal of Immunology

The data are comprehensive. The MEL5 TCR (raised against the WT peptide EAAGIGILTV) had higher affinity (~2x by SPR) for the WT peptide compared to the heteroclitic. Additionally, thermoodynamic analysis revealed distinct energetic footprints, with the WT peptide being more typical (enthalpically driven).

This group previously solved the solved the MHC-peptide-TCR structure with the heteroclitic peptide, here they report the corresponding structure with the WT peptide. Comparison of the two (and the corresponding unliganded pMHC structures) reveals the WT peptide (but not the heteroclitic) is 'pulled' away from the peptide groove enabling additional interactions which explain the increased affinity over the heteroclitic peptide.

Overall, this work provides one mechanism whereby heteroclitic peptides (with enhanced MHC binding) may be differentially 'seen' by the TCR repertoire. Indeed this feature (peptide positioned 'pulled' away from the MHC) of TCR recognition is, to my knowledge, has not been reported before adding another, interesting facet to TCR recognition.

Author Response: We thank the reviewer for their extremely positive feedback.

Reviewer1 comment : If possible, some clarification on how TCR binding of EAA and ELA are thermodynamically different (entropy/enthalpy).

Author Response: We have added some extra detail on the thermodynamic implications to our discussion (lines 188-195).

Reviewer 1 comment: As the 2 unliganded pMHC structures are very similar (peptide seated in groove) - what does this mean for MHC on the cell surface - is there wobble in/out of the groove?

Author Response: Whether the EAA peptide ‘wobbles’ in and out of the groove is a matter of speculation. However, we and others have demonstrated that the A2-EAA complex is less stable than the A2-ELA peptide (this is covered and referenced in the introduction and discussion). Thus, we speculate that A2-EAA undergoes a greater degree of flexibility at the cell surface, and that the lower stability probably means that A2-EAA would be present at a lower concentration at the cell surface compared to A2-ELA. This could impact on the sensitivity of A2-EAA specific T-cells, reducing their ability to target and destroy melanoma cells expressing this epitope (explaining the clinical findings). The higher surface density of ELA peptide compared to EAA peptide is likely to combine with its non-self structure to produce the very strong responses that are elicited by the ELA peptide in vivo and in vitro.

European Journal of Immunology

Reviewer 1 comment: Does EAA also stimulate T cells which recognise this (dominant?) form of the complex? How do they account for the low reactivity of T cells raised to ELA to EAA? In this setting, EAA in the right conformation (seen in crystal structures) should be available on the cell surface.

Author Response: It is important to remember that the ELA peptide binds to HLA A2 much better than EAA. Thus two factors are at play when these ligands are compared: (1) a difference in antigen density at the cell surface and (2) potential differences in structure due to the flexibility shown here. These factors are likely to combine to explain how most cells raised against ELA peptide do not crossreact with high sensitivity to the lower surface density expected with EAA peptide.

Reviewer 1 comment: Is it also to do with ligand stability/density on the surface?

Author Response: As described in the answer above, we believe that antigen density at the cell surface is likely to be an important factor.

Reviewer 1 comment: -In this example, EAA (used to raise MEL5) is the WT and ELA is the heteroclitic peptide. As the authors stress, it is patient responses raised to the heteroclitic peptide which can cross-react poorly with the WT potentially accounting for the poor clinical outcome. This study looks at the reverse situation - poor recognition of the heteroclitic peptide in comparison with the WT by a TCR raised against the WT. The authors should comment on this. Would it have been more useful to look at the former, problematic situation?

Author Response: There is some confusion here. The MEL5 TCR was actually raised against A2-ELA, but happens to recognise A2-EAA with better sensitivity. However, because MEL5-like T-cells are not likely to be very common, vaccination with A2-ELA probably selects T-cell clones more similar to DMF4 and DMF5 that do not recognise A2-EAA very well.

Reviewer 1 comment: Minor point- Abstract: has the ELA peptide really been used in 'hundreds of studies' ? If not, 'many studies' better.

Author Response: The ELA peptide is the best-studied T-cell tumour antigen and is the only recognised model for studying human T-cell priming. As a result, this system has certainly been used in more than a hundred studies. Nevertheless, there is no easy way to count exactly how many studies have used this system to study T-cell priming so we have changed the abstract as suggested.

RESPONSE TO REVIEWER 2:

European Journal of Immunology

Reviewer 2 comment: The manuscript of Madura et al presents extremely convincing and novel structural and biophysical results regarding the inefficient recognition of heteroclitic peptides by TCRs. The study is very well performed and the results are in my opinion clear-cut. The manuscript is well written and to the point.

Besides some minor points regarding the presentation of the crystallography statistics (see below), and some references that should be added in order to enhance the readers understanding of this very sensitive recognition, I think that the quality of the presented work, and the importance of their implications renders this manuscript of high interest to the community

Author Response: We thank the reviewer for their positive comments about importance of this study and the technical quality of our data and analyses.

Reviewer 2 comment : The results obtained in this study about the differential thermodynamic signature used by the TCR for recognizing the MHC molecule in complex with slightly different peptides are important (lines 87-97) and should be compared and discussed in regards to the results included in the publication by Allerbring et al, EJI 2013

Author Response: We have included this reference and additional discussion on line 195.

Reviewer 2 comment: Furthermore, the discussion section which is quite clear and precise should include a comment of the results presented in van Stipdonk et al, Cancer Research 2009 where similar results regarding the increased recognition of a heteroclitic version of the melanoma-associated gp100-derived epitope which is obtained only upon keeping the most profound structural mimicry and the altered peptide version.

Author Response: We have included this reference and additional discussion on line 166.

Reviewer 2 Comment 3: The authors should add information about the highest resolution shell.
It is also important that a specific section entitled 'analysis of B factors' is added before the Wilson B factors. Here the authors must add the average B values for the model. Finally the authors should provide a thorough explanation for the overall ESU based on maximum likelihood value.

Author Response: We have added information about the highest resolution shell and a summary of the B factor analysis to table 1 as suggested by the reviewer. We detail the analysis of B factors in the table below. These details are not normally included in a study of this type. However, we are happy to add this data as an additional supplementary table if the journal thinks this is appropriate.

Concerning the request for a thorough explanation of overall ESU based on maximum likelihood value: During refinement REFMAC reports the overall ESU based on maximum likelihood value. We have reported it in table 1 from the PDB header.

Results of B factor analysis

Chain Atoms Average Bs rms B
Main chain Side chain All atoms Main chain Side chain
A 2263 71.030 74.457 72.779 1.973 3.924
B 837 75.927 81.469 78.820 2.712 4.154
C 66 37.522 39.357 38.245 1.599 3.889
D 1498 72.896 77.574 75.151 2.432 4.251
E 1926 60.377 65.074 62.694 2.398 4.203
G 16 n/a n/a 65.154 1.850 n/a
W 20 n/a n/a 43.712 n/a n/a
Protein atoms 6590 70.792
All atoms 6626 68.506 72.871* 70.697 2.292 4.102

*Includes waters and solvent molecules

Second Editorial Decision – 23 October 2014

Dear Prof. Sewell,

It is a pleasure to provisionally accept your manuscript entitled "Structural basis for ineffective T-cell responses to MHC anchor residue improved ‘heteroclitic’ peptides" for publication in the European Journal of Immunology. For final acceptance, please follow the instructions below and return the requested items as soon as possible as we cannot process your manuscript further until all items listed below are dealt with.

Please note that EJI articles are now published online a few days after final acceptance (see Accepted Articles: http://onlinelibrary.wiley.com/journal/10.1002/(ISSN)1521-4141/accepted). The files used for the Accepted Articles are the final files and information supplied by you in Manuscript Central. You should therefore check that all the information (including author names) is correct as changes will NOT be permitted until the proofs stage.

Peer review correspondence

We look forward to hearing from you and thank you for submitting your manuscript to the European Journal of Immunology.

Yours sincerely,
Karen Chu

on behalf of Prof. David Gray

Dr. Karen Chu
Editorial Office
European Journal of Immunology
e-mail: ejied@wiley.com
www.eji-journal.eu
